# Editorial: Role of hormones and bioactive components in breast milk on development of metabolic, neural and behavioral systems in offspring

**DOI:** 10.3389/fendo.2023.1261078

**Published:** 2023-08-02

**Authors:** Angel I. Melo, Kurt L. Hoffman

**Affiliations:** Centro de Investigación en Reproducción Animal, CINVESTAV, Laboratorio Tlaxcala, Universidad Autónoma de Tlaxcala, Tlaxcala, Mexico

**Keywords:** breastmilk, growth and development, pesticides, critical periods (CPs), bioactive compounds

Early life experiences in mammals shape developmental processes in most physiological systems: in altricial mammals such as rodents and primates such experience interacts with genetic backround from the fetal stage to the first postnatal days (rodents) or months (humans). Although each species is unique and has its own developmental scheme, one can propose three critical periods of development during which external stimuli – experience – can positively or negatively modify genetically-programmed developmental processes through epigenetic mechanisms: the prenatal period, postnatal/pre-weaning period, and the juvenile/adolescence period. Among species, the timing and duration of these three main critical periods differ greatly. Moreover, different physiological systems (e.g., neural, metabolic, immune) often have distinct temporal windows of sensitivity within each of the critical periods.

During pregnancy (prenatal intrauterine period) the effects of the environmental factors depend on the identity of the factor as well as the specific prenatal stage of exposure to that factor (germinal, embryonic, or fetal). For example, the neural tube develops into the nervous system during the fetal stage (in humans, from the ninth week until birth) and the neurons begin to migrate to their location and start establishing connections. Therefore, exposure to certain environmental factors during this period could positively or negatively affect the developing brain, depending on the nature of the factor. After birth, the immature infant advances to a new developmental phase where he/she receives nutrition from breastmilk and crucial sensory and social cues (primarily from the mother, but also from siblings, father and other conspecifics) that are necessary to achieve physiological independence [See (1, 2)].

Breastmilk has attracted the scientific attention since the 1930´s, but studies have mainly focused on its nutritional function: the chemical structures of the macronutrients (carbohydrates, proteins, fats) and their functions on the growth and development of the infant. But the introduction of more sophisticated analytical technologies like the differential ultracentrifugation method has allowed the identification of a plethora of specific bioactive components in breastmilk, and has provided new insights into the effects of breastfeeding on the development of the physical and mental wellbeing of the infant and the mother.

Breastmilk is an important source of macro- and micronutrients for the growth, development, and maturation of the mammalian infant. Breastmilk is also an important source of hormones (e.g., leptin, insulin, ghrelin, adiponeptin, corticoids, melatonin), growth factors, exosomas, microRNAs, probiotics, lipophilic micronutrients (e.g., vitamins), proteins, pluripotent stem cells, leucocytes, immunoglobulins, and polar metabolites (e.g., oligossacharides) microbiota and other bioactive compounds. These milk-borne factors contribute to the composition of intestinal flora, and impact on the development of reproductive, immunologic, metabolic, and neural systems. Furthermore, breastmilk has anti-inflammatory, antioxidant, antimicrobial, anticarcinogenic and immunological properties. The concentrations of these components in milk changes during lactation according to the needs and stage of development of the infant, being significantly higher in early milk or colostrum [See (3–7)]. However, breastmilk also can contain harmful substances that can be transmitted to the infants, including drugs, toxins, contaminants, microplastics, and pathogens (virus, bacteria, fungus) that can negatively impact the growth, maturation, and health of the offspring [See (8, 9)].

The intention of this Research Topic was to integrate the most recent findings related to breastmilk components that directly or indirectly affect the infant’s development and growth, as well their mental and physical health. In this Research Topic, Qi et al. reviewed how the most common organochlorine pesticides (OCPs) and some newer ones such as dichlorodiphenyltrichloroethane (DDT), methoxychlor (MXC), hexachlorobenzene (HCB), pollutants found in the environment, can accumulate in breastmilk and be passed to the infant, possibly having negative effects on the infant’s health. In addition, the authors update the discussion of the most commonly used methods for the management of these contaminants, with the intention of reducing the negative effects on both the mother and the babies.

Breast milk composition changes throughout lactation, depending on the maternal diet, environmental contamination, emotional state (e.g., stress), parity and lactation stage [See (3, 10)]. Thus, in this Research Topic Ramiro-Cortijo et al. studied how maternal body composition and diet during lactation influence the levels of ghrelin, leptin, resistin, insulin, peptide YY and gastrointestinal peptide in breastmilk, as well how these affect the neonatal growth of preterm infants. They found that: 1) women with preterm labor had a lower level of ghrelin in the breastmilk, which was associated with maternal fat store and diet, and 2) the growth of premature babies was positively associated with breastmilk ghrelin, but in term infants the growth was positively associated with insulin and negatively with peptide YY.

The composition of the breastmilk also depends on the mother’s diet, health, or disease (e.g., gestational diabetes mellitus; GDM), chronic stress, as well the pattern of breastfeeding; exclusive, bottle-nursing, or mixed (11, 12). Qian et al. studied how breastfeeding develops in mothers with GDM, and how GDM was associated with long-term health in both the mother and the infant. They found that the mother rarely considered their health condition of GDM for their decision to breastfeed or not, primarily because they did not have reliable sources of information, and they believed that the disease was a minor and transient health problem, without considering the long-term risk of GDM and the possible protective effects of breastfeeding for them and their babies.

Finally, Kaneko et al., as part of the Japan Environment and Children’s Study, examined the association between maternal total cholesterol (TC) in mid-pregnancy with the failure to achieve normal weight (“No catch-up”) by the age of 3, in full-term infants that were born small for their gestational age. They found an association of high maternal TC at mid-pregnancy with altered development in these infants. These results are in agreement with pre-clinical findings and suggest that a high-fat diet during pregnancy can disturb the growth and development of the offspring (13).

The works of this Research Topic emphasize the importance of maternal nutrition during pregnancy on the infant’s development, as well as the benefits of breastfeeding for both the mother and the infant. Although breastmilk is a crucial source of nutrients, hormones, and a multitude of other factors important for infant development, it can also be a source of exposure to environmental contaminants. Given the increasingly contaminated modern world, it is urgent that more research is carried out on the developmental effects of the latter.

## Author contributions

AM: Writing – original draft, Writing – review & editing. KH: Writing – review & editing
